# Retrospective analysis of the upper airway anatomy and Sella turcica morphology across different skeletal malocclusions: a computerized technique

**DOI:** 10.1186/s12903-024-04867-6

**Published:** 2024-09-19

**Authors:** Anand Marya, Samroeng Inglam, Adrien Dagnaud, Sujin Wanchat, Prasitthichai Naronglerdrit, Horn Rithvitou, Nattapon Chantarapanich

**Affiliations:** 1 Faculty of Dentistry, Thammasat University, Klong Luang, Pathumthani, 12120 Thailand; 2 ECAM LaSalle, Lyon, 69 321 France; 3 Department of Mechanical Engineering, Faculty of Engineering at Sriracha, Kasetsart University, Sriracha, Chonburi, 20230 Thailand; 4 Department of Computer Engineering, Faculty of Engineering at Sriracha, Kasetsart University, Sriracha, Chonburi, 20230 Thailand; 5 Faculty of Dentistry, University of Puthisastra, Phnom Phen, 12211 Cambodia

**Keywords:** Upper airway, Sella turcica, Skeletal class, CBCT, Correlation, Gender

## Abstract

**Objective:**

This study aimed to investigate the normal volumetric space and variations in the measurements of different landmarks in adults with different skeletal relations of the maxilla and the mandible based on CBCT data. The study also analyses these landmarks to locate any correlations.

**Background:**

Numerous studies in orthodontics have found a relationship between orthodontic treatment and changes in the anatomy and function of the airway. Severe changes in airway morphology can cause breathing difficulties, lower quality of life, and even result in life-threatening conditions such as obstructive sleep apnoea. Consequently, orthodontic diagnosis and treatment planning require a thorough understanding of the airway space and its function.

**Methods:**

The present retrospective study was conducted using CBCT records of 120 adult patients, containing 40 samples of each skeletal class (20 males and 20 females). The boundaries were defined for the 3 major regions: the nasopharynx, the oropharynx, and the hypopharynx. Various measurements were recorded across these regions, as well as selective cephalometric landmarks. The obtained data was used to calculate average and standard deviation, while regression analysis was used to evaluate correlations and t-test was used to test statistical significance of gender differences.

**Results:**

The results demonstrate that skeletal Class III individuals exhibit a reduced airway volume in the nasopharynx compared to other groups, whereas skeletal Class II individuals displayed a diminished airway volume in the hypopharynx. A strong correlation was observed for Sella turcica parameters. There were no significant differences in skeletal parameters across genders. Nasopharynx cavity volume demonstrated significant differences between skeletal Class I–Class III as well as between skeletal Class II–Class III. Hypopharynx cavity volume also demonstrated significant differences between skeletal Class I–Class II and between skeletal Class II–Class III.

**Conclusion:**

The major findings are the presence of a reduced nasopharyngeal volume in skeletal Class III malocclusions while skeletal Class II individuals displayed a diminished hypopharyngeal volume, making these critical areas to consider during the diagnostic and orthodontic treatment planning stages. This study also revealed a consistent correlation between Sella turcica parameters across various facial skeletal profiles, with skeletal Class II patients exhibiting a distinct pattern and skeletal Class I and Class III demonstrating an average relationship.

## Introduction

The airway space in the body includes the area from above the vocal cords to the nasal and the oral cavity [1, 2]. Medical professionals, such as ENT specialists, maxillofacial surgeons, and orthodontists, have recently paid attention to changes in the pharyngeal airway morphology resulting from various management modalities [3]. Severe changes in airway morphology can cause breathing difficulties, lower quality of life, and even result in life-threatening conditions such as obstructive sleep apnoea (OSA) [4]. OSA is a condition in which breathing is blocked or inadequate during sleep and affects between 2 and 7% of adults [5]. However, OSA often goes undiagnosed due to its lengthy and expensive diagnostic process [6].

Several techniques are currently being employed for the diagnosis of OSA. The more conventional method, polysomnography, remains the gold standard in OSA diagnosis. Doctors and sleep specialists use the apnoea-hypopnea index (AHI) to determine the severity of sleep apnoea. However, this method is costly, takes a lot of time, and AHI results are not always reliable [7]. Researchers are currently working on developing new techniques that use image modalities to reflect the status of the upper airway directly [8]. This could potentially make the diagnosis of sleep apnoea faster and more accurate. While X-ray cephalometry was developed for craniofacial morphology assessment, the complexity of the airways could not be entirely determined using 2-dimensional records [9]. Over the years, several 3-dimensional (3D) imaging techniques such as computed tomography (CT) and cone beam CT (CBCT) have been developed to evaluate changes in the airways from the tip of the nose to the upper end of the trachea [10].

CBCT produces high-quality and dependable reconstructions of the 3D morphology of airway structures. Compared to other methods, it has the advantages of 3D analysis, lower exposure to radiation, and short scanning time [11]. This is why CBCT has been widely accepted by clinicians to conduct airway structure analysis. CBCT enables accurate analysis of cross-sectional areas and volumetric regions side-by-side [12]. Moreover, using CBCT can help build an anatomical profile and determine the pressure and air flow in different areas. Numerous studies in orthodontics have found a relationship between orthodontic treatment and changes in the anatomy and function of the airway [13]. Orthodontists use standard procedures to correct jaw discrepancies, but these procedures can affect soft tissues, including the pharyngeal airway, which may lead to obstructive sleep apnoea (OSA) [14]. Researcher has also found a link between a narrower upper airway size and the distance of incisor retraction [14]. Consequently, orthodontic diagnosis and treatment planning must utilize a thorough understanding of the airway space and its function.

In the past few years, many studies have been conducted across different malocclusions with a focus on the airway. Nonetheless, many of these studies have limitations in terms of their findings since they focus on comparing pre- and post-treatment results to evaluate treatment related changes across the airways [15–17]. Meanwhile, other studies compared airway morphology across different skeletal classes, but these have considered the airway as a whole rather than divided into smaller components such as the nasopharynx, oropharynx, and the hypopharynx [18–20]. The major limitation of these studies is that while they were able to locate changes in the upper pharyngeal airway with different skeletal malocclusions, they were unable to localise it to a particular region or area in the airway.

In this study, the primary objective was to find out the normal volumetric space and variations in the measurements of different anatomical parameters in adults with different skeletal relations of the maxilla and the mandible by means of CBCT analysing software. The functional and anatomic variations of adult patients with ideal Class I, Class II, and Class III skeletal relationships were evaluated. Evaluation of the airway space in the various regions of the pharynx was performed following 3D modelling of CBCT images from each skeletal class. The length and volume of various anatomical structures were measured across the nasopharynx, oropharynx, and hypopharynx. Moreover, the study also identified and evaluated the airway space parameters so they could be incorporated into treatment planning. To avoid compromising the respiratory airway after orthodontic treatment, these parameters must be carefully considered during the planning phase.

## Materials and methods

### Subjects and inclusion

The study protocol was approved by the Kasetsart University Research Ethics Committee of the Kasetsart University, Sriracha Campus (Study Code: KUREC-SRC66/029), Thailand. The present retrospective study was conducted using CBCT records from the existing subject database at the Faculty of Dentistry, University of Puthisastra, Phnom Penh, Cambodia, which was scanned by CBCT machine (Vatech PAX i3D Green, VATECH Co., Ltd., South Korea). The patients were classified by measuring the ANB angle using the lateral cephalometric record derived from the CBCT, i.e. Class I (ANB: 0–4 degrees), Class II (ANB: > 4 degrees), and Class III (ANB: <0 degree). A CBCT of each skeletal class was selected according to following criteria:

1. CBCT data of adult patients without any craniofacial anomalies, defects, or affected by syndromes;
2. CBCT data of adults with aged 18 years or older;
3. CBCT of adults without any systemic health problems at the time of taking records; and.
4. CBCT of adults without history of tonsillectomy, having enlarged adenoids at the time of records.

In total, CBCT data from 120 adult patients and including all three classes were selected for this study. Each skeletal class contained 40 patients’ data, with 20 males and 20 females.

### CBCT data processing and anatomical parameters

The CBCT data was formatted in Digital Imaging and Communications in Medicine (DICOM) files which were later imported into 3D Slicer (Slicer.org) [21] for image segmentation and visualization. The 3D reconstruction was performed by thresholding the airway. The 3D model of the airway was divided into three major regions, nasopharynx, oropharynx, and hypopharynx. Each region was segmented according to the definition in Table 1. The thresholding result provided the 3D stereolithography (STL) model of airway segments. These models were used to measure the anatomical distance or volume using Computer Aided Design (CAD) software (VISI, Hexagon AB, Sweden) according to the following anatomical measurements:

1. Nasopharynx cavity volume (NCV), unit: cm^3^,
2. Oropharynx cavity volume (OCV), unit: cm^3^,
3. Hypopharynx cavity volume (HCV), unit: cm^3^,
4. Length of the soft palate (LSP), unit: mm,
5. Distance between the soft palate tip to the posterior wall of pharynx (DSP), unit: mm,
6. Distance between the epiglottis tip to the posterior wall of the pharynx (DEP), unit: mm,
7. Sella turcica diameter (SDI), unit: mm,
8. Sella turcica length (STL), unit: mm, and
9. Sella turcica depth (SDE), unit: mm.

**Table 1:** Boundary definition for the three pharyngeal regions

Airway region	Anterior boundary	Posterior boundary	Superior boundary	Inferior boundary
Nasopharynx	Line extending from S to the PNS	Line extending from S to the tip of the odontoid process	Line extending from S to the tip of the odontoid process	Line extending from PNS to the tip of the odontoid process
Oropharynx	Line extending from PNS to the tip of the epiglottis	Line extending from the tip of the odontoid process to the posterior superior border of CV4	Line extending from PNS to the tip of the odontoid process	Line extending from the base of the epiglottis to the posterior superior border of CV4
Hypopharynx	Line extending from the base of the epiglottis to the inferior border of the symphysis	Line extending from the posterior–superior corner of CV4 to the posterior–inferior corner of CV4	Line extending from the base of the epiglottis to the posterior superior border of CV4	Line extending from the posterior–inferior corner of CV4 to the inferior border of the symphysis

### Analysis

Average and standard deviation were calculated for each of the anatomical parameters. The data was also plotted to visualise variation among different skeletal classes. The data was further analysed for correlation based on linear regression analysis among different parameter pairs. The correlation was represented by coefficient of determination (r^2^) and *p*-value. A t-test was also performed (MS Excel, Microsoft Corporation, US) to analyse whether any differences existed between the measurements obtained from the male and female patients as well as across skeletal classes.

## Results

**Fig. 1:**
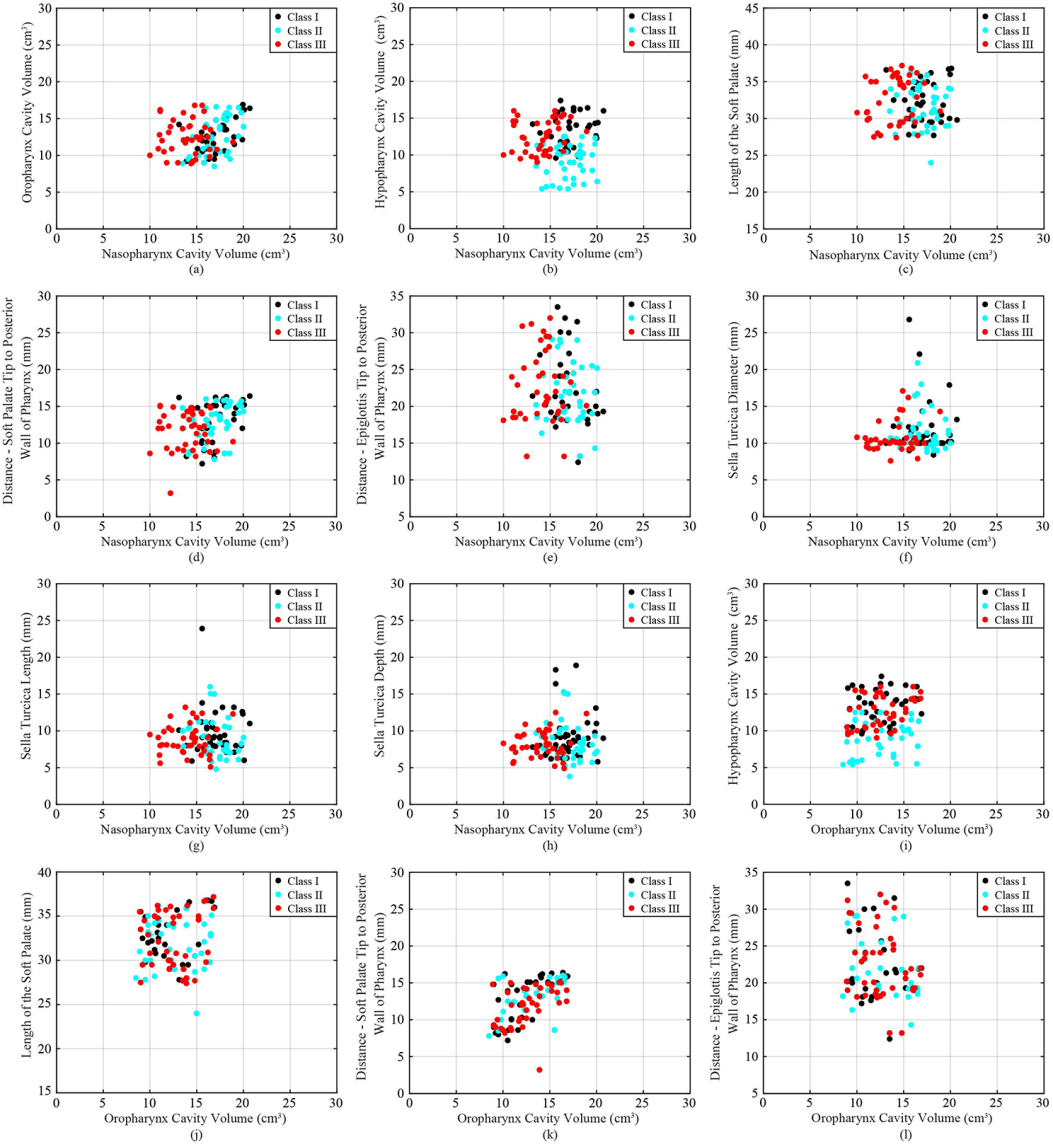
Scatter plot demonstrating relation and distribution between (**a**) NCV-OCV, (**b**) NCV-HCV, (**c**) NCV-LSP, (**d**) NCV-DSP, (**e**) NCV-DEP, (**f**) NCV-SDI, (**g**) NCV-STL, (**h**) NCV-SDE, (**i**) OCV-HCV, (**j**) OCV-LSP, (**k**) OCV-DSP, and (**f**) OCV-DEP of each skeletal class

**Fig. 2:**
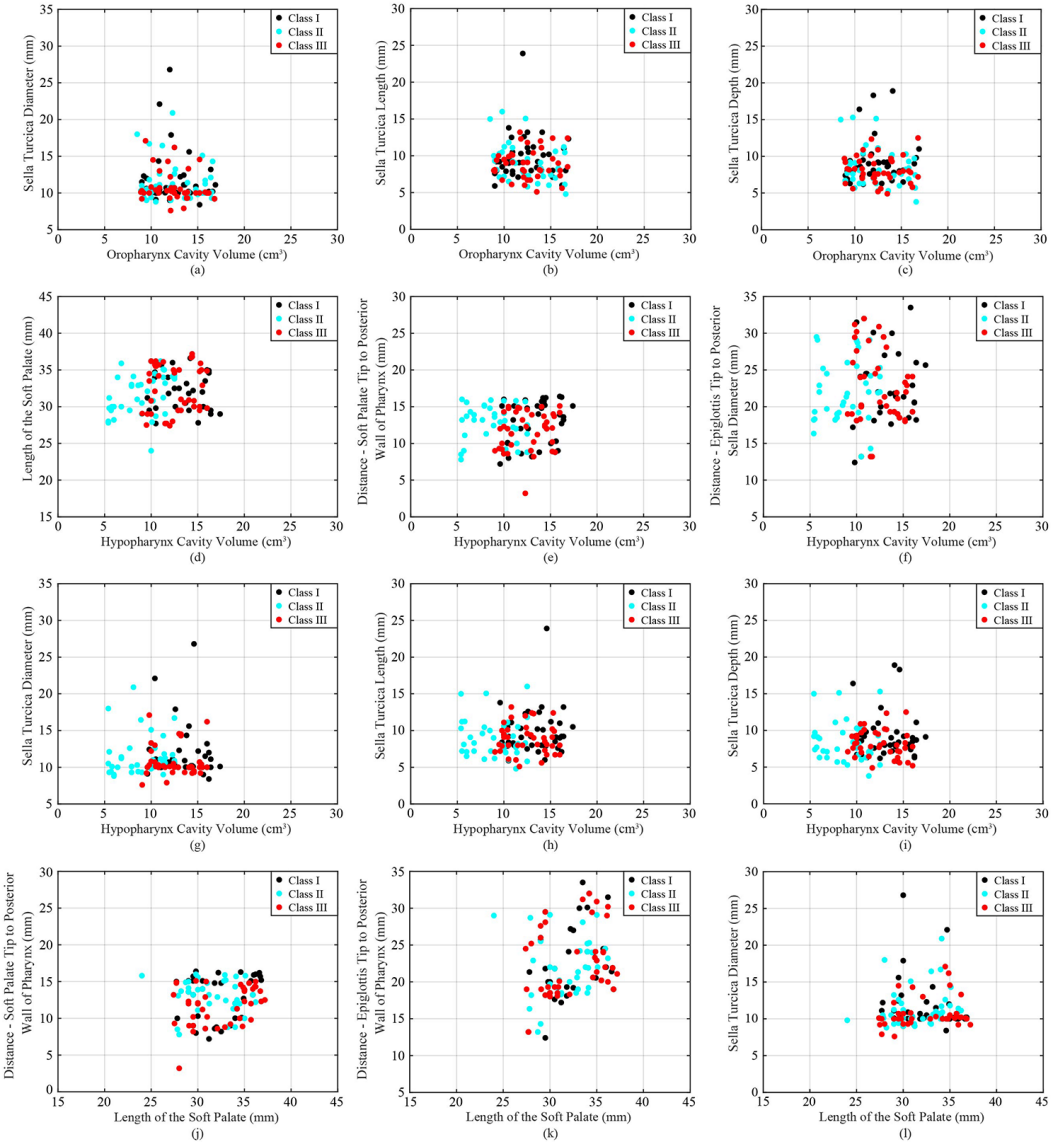
Cluster plot demonstrating relation and distribution between (**a**) OCV-SDI, (**b**) OCV-STL, (**c**) OCV-SDE, (d) HCV-LSP, (**e**) HCV-DSP, (**f**) HCV-DEP, (**g**) HCV-SDI, (**h**) HCV-STL, (**i**) HCV-SDE, (**j**) LSP-DSP, (**k**) LSP-DEP, and (**f**) LSP-SDI of each skeletal class

**Fig. 3:**
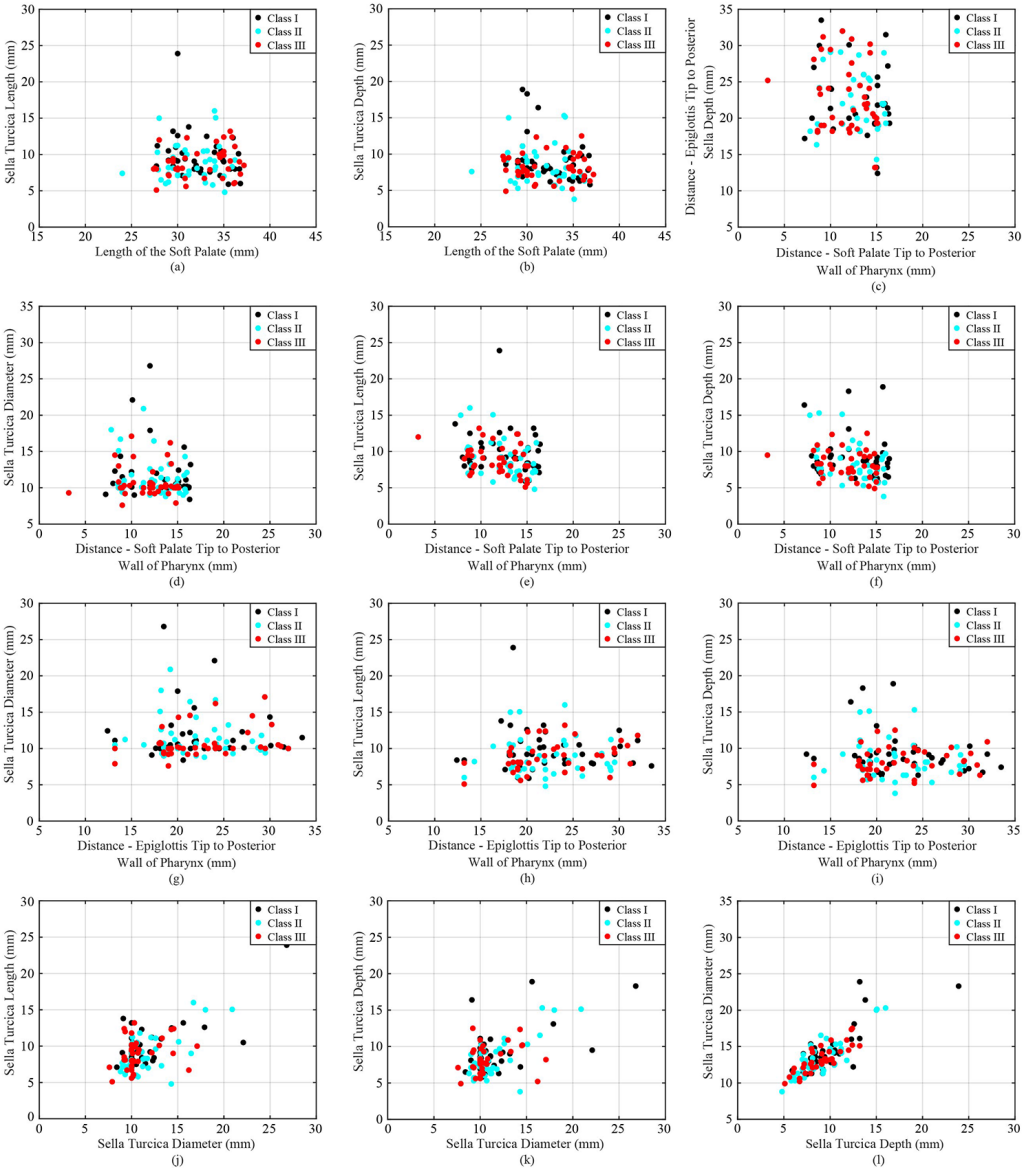
Cluster plot demonstrating relation and distribution between (**a**) LSP-STL, (**b**) LSP-SDE, (**c**) DSP-DEP, (**d**) DSP-SDI, (**e**) DSP-STL, (**f**) DSP-SDE, (**g**) DEP-SDI, (**h**) DEP-STL, (**i**) DEP-SDE, (**j**) SDI-STL, (**k**) SDI-SDE, and (**f**) SDE-SDI of each skeletal class

Table 2 presents the average along with standard deviation of each anatomical parameter for each skeletal class. The values are reported separately for each gender (male and female) and both genders combined. In addition, Figs. 1, 2 and 3 include a scatter plot showing correlation between two skeletal parameters as well as data visualization of each class. Remarkably, individuals with a skeletal Class III malocclusion exhibit a reduced cavity volume in the nasopharynx compared to other groups (Fig. 1(a)), while Class II individuals display a diminished cavity volume in the hypopharynx (Fig. 1(b)). The DEP varies significantly across different skeletal profiles (Fig. 2(k)). For individuals with Class III skeletal relationships, their SDI demonstrates a notably low range, with many values hovering around 10 mm. In contrast, individuals with Class I show more diverse and scattered Sella related values (Fig. 2(g)). It can be observed that regardless of the facial skeletal profile, there seems to be a systematic relationship among SDI, STL, and SDE, while this relationship also seems particularly characterised for Class III (Fig. 3(j), 3(k), and 3(f)).

**Table 2:** Average values and the standard deviation (SD) of each skeletal parameter

Parameters	Skeletal class I average (SD)			Skeletal class II average (SD)			Skeletal class III average (SD)		
	Male (*n* = 20)	Female (*n* = 20)	Combined (*n* = 40)	Male (*n* = 20)	Female (*n* = 20)	Combined (*n* = 40)	Male (*n* = 20)	Female (*n* = 20)	Combined (*n* = 40)
NCV (Unit: cm^3^)	17.43 (1.67)	16.75 (1.84)	17.09 (1.74)	16.80 (1.73)	17.18 (1.77)	16.99 (1.74)	13.53 (1.93)	14.46 (2.04)	13.99 (2.02)
OCV (Unit: cm^3^)	12.49 (2.58)	12.18 (1.94)	12.33 (2.23)	12.50 (2.75)	12.64 (2.49)	12.57 (2.59)	12.37 (2.55)	12.75 (2.12)	12.56 (2.32)
HCV (Unit: cm^3^)	13.29 (2.54)	13.18 (2.09)	13.23 (2.27)	8.99 (2.31)	9.06 (2.37)	9.03 (2.31)	12.43 (2.29)	12.62 (2.12)	12.52 (2.18)
LSP (Unit: mm)	32.59 (3.00)	32.13 (2.50)	32.36 (2.70)	31.24 (2.95)	31.83 (2.51)	31.53 (2.72)	32.14 (3.29)	32.67 (3.14)	32.40 (3.19)
DSP (Unit: mm)	12.76 (2.92)	12.64 (3.04)	12.70 (2.90)	12.74 (2.75)	12.52 (2.26)	12.63 (2.49)	12.01 (2.24)	11.56 (3.04)	11.79 (2.64)
DEP (Unit: mm)	22.94 (5.32)	21.90 (4.53)	22.42 (4.84)	21.96 (4.16)	21.99 (4.23)	21.97 (4.14)	22.59 (5.08)	22.63 (4.64)	22.61 (4.80)
SDI (Unit: mm)	11.83 (4.48)	11.40 (2.27)	11.61 (3.47)	13.20 (7.78)	11.49 (2.87)	12.34 (5.85)	10.22 (0.84)	11.32 (2.72)	10.77 (2.06)
STL (Unit: mm)	9.69 (3.77)	9.83 (2.11)	9.76 (2.98)	10.87 (8.89)	8.81 (2.27)	9.84 (6.49)	8.70 (1.28)	9.04 (2.58)	8.87 (2.01)
SDE (Unit: mm)	8.82 (2.60)	9.53 (3.27)	9.17 (2.90)	10.06 (7.63)	8.19 (2.29)	9.13 (5.64)	8.08 (1.31)	8.29 (2.25)	8.18 (1.82)

**Fig. 4:**
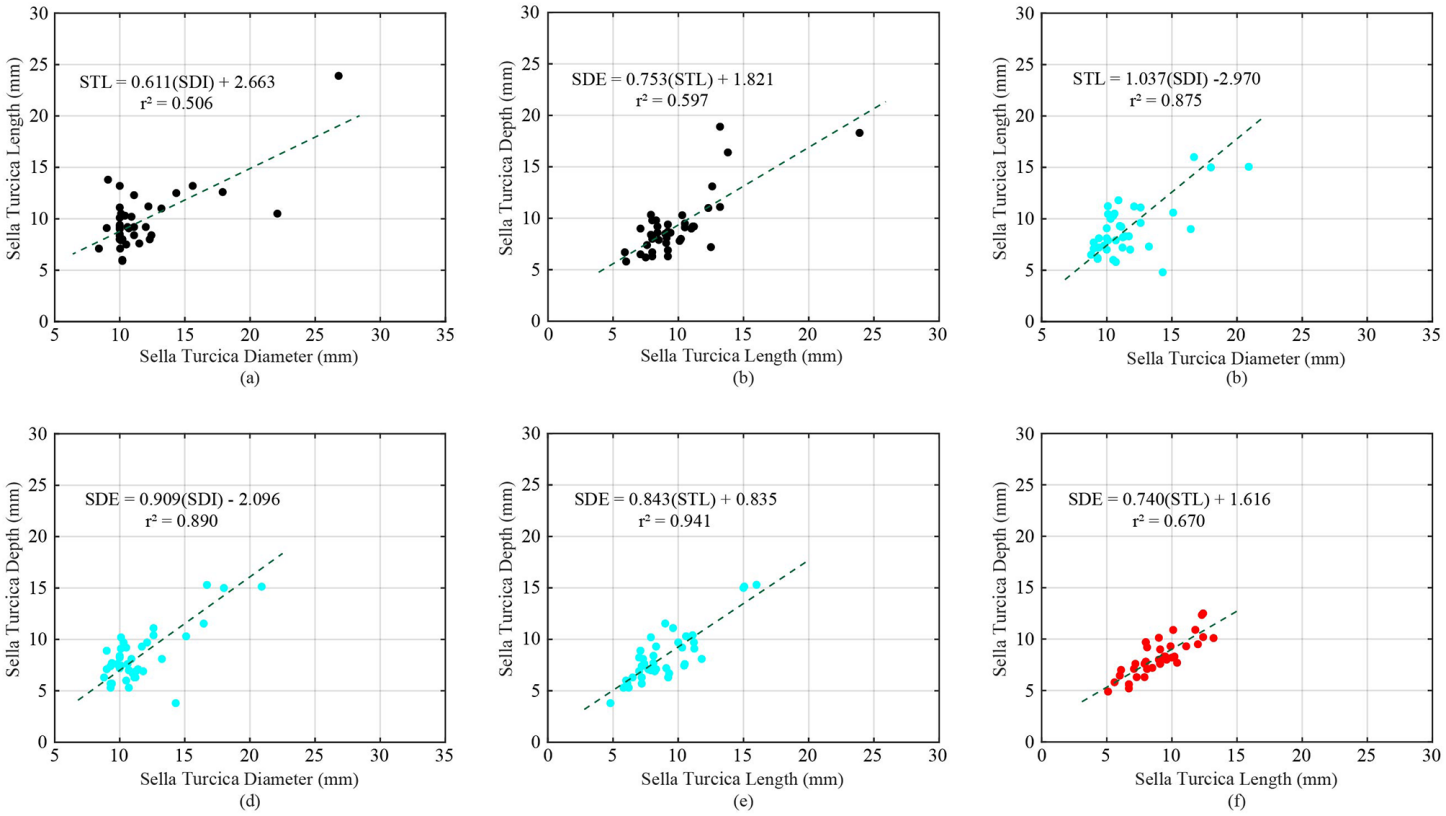
Strong coefficient of determination (r^2^) pair (**a**) Class I: SDI-STL, (**b**) Class I: STL-SDE, (**c**) Class II: SDI-STL, (**d**) Class II: SDI-SDE, (**e**) Class II: STL-SDE, and (**f**) Class III: STL-SDE

The coefficient of determination (r^2^) between pair of each parameter of each skeletal class are presented in Tables 3, 4 and 5. Strong corelations were observed for SDI-STL of Class I (r^2^ = 0.506, *p-value* = 2.693E-7), STL-SDE of Class I (r^2^ = 0.597, *p-value* = 5.298E-9), SDI-STL of Class II (r^2^ = 0.875, *p-value* = 8.837E-19), SDI-SDE of Class II (r^2^ = 0.890, *p-value* = 8.362E-20), STL-SDE of Class II (r^2^ = 0.941, *p-value* = 6.845E-25), and STL-SDE of Class III (r^2^ = 0.670, *p-value* = 1.081E-10). (See Fig. 4).

**Table 3:** Coefficient of determination (r²) and *p*-value (in bracket) for each pair of skeletal parameters in Class I individuals

Parameter	NCV	OCV	HCV	LSP	DSP	DEP	SDI	STL	SDE
NCV		0.395 *(1.430E-5)*	0.008 *(0.573)*	2.961E-4 *(0.916)*	0.171 *(0.008)*	0.083 *(0.071)*	2.57E-6 *(0.992)*	0.002 *(0.800)*	0.007 *(0.598)*
OCV			0.021 *(0.371)*	0.018 *(0.404)*	0.454 *(1.910E-6)*	0.058 *(0.135)*	0.003 *(0.745)*	0.002 *(0.782)*	0.007 *(0.618)*
HCV				0.027 *(0.313)*	0.049 *(0.170)*	0.008 *(0.594)*	1.264E-4 *(0.945)*	0.010 *(0.533)*	0.006 *(0.630)*
LSP					0.054 *(0.151)*	0.116 *(0.032)*	0.031 *(0.280)*	0.106 *(0.040)*	0.111 *(0.035)*
DSP						0.033 *(0.260)*	0.012 *(0.497)*	0.027 *(0.310)*	0.009 *(0.550)*
DEP							0.005 *(0.668)*	0.008 *(0.576)*	0.043 *(0.200)*
SDI								0.506 *(2.693E-7)*	0.292 *(3.147E-4)*
STL									0.597 *(5.298E-9)*
SDE									

**Table 4:** Coefficient of determination (r²) and *p*-value (in bracket) for each pair of skeletal parameters in Class II individuals

Parameter	NCV	OCV	HCV	LSP	DSP	DEP	SDI	STL	SDE
NCV		0.285 *(3.889E-4)*	0.025 *(0.331)*	5.831E-5 *(0.963)*	0.186 *(0.005)*	0.016 *(0.441)*	0.001 *(0.852)*	0.008 *(0.586)*	0.007 *(0.607)*
OCV			0.017 *(0.417)*	0.002 *(0.764)*	0.252 *(0.001)*	0.106 *(0.040)*	0.007 *(0.607)*	7.291E-5 *(0.958)*	3.306E-6 *(0.991)*
HCV				0.146 *(0.015)*	0.003 *(0.732)*	0.006 *(0.643)*	0.002 *(0.812)*	0.002 *(0.812)*	0.004 *(0.684)*
LSP					0.007 *(0.598)*	0.006 *(0.638)*	0.001 *(0.821)*	2.123E-4 *(0.929)*	0.009 *(0.558)*
DSP						0.021 *(0.377)*	0.004 *(0.691)*	0.009 *(0.555)*	0.011 *(0.522)*
DEP							0.005 *(0.657)*	2.198E-4 *(0.928)*	0.002 *(0.802)*
SDI								0.875 *(8.837E-19)*	0.890 *(8.362E-20)*
STL									0.941 *(6.845E-25)*
SDE									

**Table 5:** Coefficient of determination (r²) and *p*-value (in bracket) for each pair of skeletal parameters in Class III individuals

Parameter	NCV	OCV	HCV	LSP	DSP	DEP	SDI	STL	SDE
NCV		1.043E-4 *(0.950)*	0.037 *(0.237)*	0.029 *(0.293)*	1.179E-4 *(0.947)*	0.002 *(0.785)*	0.046 *(0.183)*	0.006 *(0.624)*	0.014 *(0.468)*
OCV			0.146 *(0.015)*	0.012 *(0.500)*	0.213 *(0.003)*	0.071 *(0.096)*	0.041 *(0.208)*	0.001 *(0.856)*	0.002 *(0.786)*
HCV				0.012 *(0.510)*	0.031 *(0.273)*	0.062 *(0.121)*	0.001 *(0.866)*	0.003 *(0.730)*	0.029 *(0.289)*
LSP					0.095 *(0.053)*	0.094 *(0.055)*	0.041 *(0.211)*	0.056 *(0.142)*	0.002 *(0.786)*
DSP						0.054 *(0.147)*	0.001 *(0.834)*	0.115 *(0.031)*	0.047 *(0.177)*
DEP							0.107 *(0.039)*	0.103 *(0.043)*	0.035 *(0.239)*
SDI								0.091 *(0.058)*	0.067 *(0.108)*
STL									0.670 *(1.081E-10)*
SDE									

For gender-based differences, there was no significant mean difference in all skeletal parameter as all *p*-values were no less than 0.05 (Table 6). Across the skeletal class, NCV demonstrated significant differences between skeletal Class I–Class III as well as between skeletal Class II–Class III. In addition, HCV also demonstrated significant differences between skeletal Class I–Class II and between skeletal Class II–Class III (Table 7).

**Table 6:** *p*-value across gender of each skeletal class for each parameter

Parameters	Skeletal class I	Skeletal class II	Skeletal class III
NCV	0.224	0.489	0.147
OCV	0.673	0.871	0.614
HCV	0.878	0.924	0.783
LSP	0.608	0.494	0.608
DSP	0.898	0.790	0.597
DEP	0.509	0.983	0.987
SDI	0.704	0.367	0.095
STL	0.879	0.327	0.596
SDE	0.454	0.305	0.720

**Table 7:** *p*-value across skeletal class for each parameter

Parameters	Skeletal class I - Skeletal class II	Skeletal class I - Skeletal class III	Skeletal class II - Skeletal class III
NCV	0.802	2.081E-10*	4.717E-10*
OCV	0.658	0.659	0.979
HCV	4.528E-12*	0.159	9.315E-10*
LSP	0.180	0.947	0.194
DSP	0.907	0.148	0.146
DEP	0.659	0.863	0.527
SDI	0.499	0.196	0.115
STL	0.946	0.124	0.372
SDE	0.962	0.074	0.318

(Remark * significant difference)

## Discussion

This study reveals a consistent correlation among Sella turcica parameters and various facial skeletal profiles, with skeletal Class II individuals exhibiting a distinct pattern and skeletal Class I and Class III showing an average relationship. Notably, specific relationships emerge for skeletal Class II in terms of Sella dimensions. From the findings, airway volume discrepancies in nasopharynx and hypopharynx were observed in skeletal Class III and skeletal Class II individuals, respectively, highlighting the impact of facial skeletal profiles on airway morphology. The variation in epiglottis-to-pharynx wall distance and Sella diameter emphasises the diversity within different skeletal classes.

At present, there is very limited literature comparing skeletal Class II patients with other skeletal classes and information in these areas is mostly very limited [22]. Schwab et al. observed that retrognathism of the maxilla and the mandible led to a narrowing of the posterior pharyngeal airway [15]. Meanwhile, Muto et al. also observed that patients with mandibular retrognathism had constriction of the oropharyngeal airway, but the analysis was conducted at the level of the base of the tongue and the soft palate [20]. The present study reveals that Skeletal Class II individuals had a constriction of the pharyngeal airway space at the level of the hypopharynx. Skeletal Class II mal-relations are characterised by a relative mandibular retrognathism and posterior rotations of the mandible. These are majorly divided into three subgroups depending upon whether these are caused due to a retrognathic mandible, prognathic maxilla, or a combination of both. Previous studies demonstrate that management of skeletal Class II individuals can help improve respiratory problems in cases with airway related problems [23, 24]. Since skeletal Class II individuals have a narrow pharyngeal airway, it can also lead to changes in the respiratory tract [25].

Ceylan et al. previously studied the pharyngeal airway across different skeletal relations using a similar sample size as the study and found that the oropharyngeal space became smaller as the ANB angle increased [25]. However, this study was conducted using 2D cephalometric records which was less precise computing compared to 3D volumetric records. By using CBCT data, this study could subdivide the upper pharyngeal space into smaller areas such as the nasopharynx, the oropharynx, and the hypopharynx. It was found that in individuals with an increased ANB angle, i.e., Class II, malocclusions there was a corresponding decrease in the hypopharyngeal space. Chokotiya et al. also explored pharyngeal airway size across different skeletal malocclusions but found no change in the dimensions of the pharyngeal airway space with a change in the ANB angle. They also found no gender related difference in the pharyngeal airway dimensions in males or females [26], which correspond well will the findings of the present study. According to Fujita and Simmons there are three major patterns of obstruction leading to sleep apnea (OSA): type I- retropalatal only, type II- retropalatal and hypopharyngeal combination and type III- hypopharyngeal only [27]. Identifying a reduced hypopharynx in affected patients could act as the initial step in the diagnosis and treatment planning stage. Many of the severely affected patients may even require hypopharyngeal surgery which has demonstrated improved outcomes in terms of managing OSA patients with a reduced hypopharyngeal volume [27, 28]. OSA has been previously associated with systemic disorders such as: renal, cardio-vascular, pulmonary and metabolic problems. In recent years there is more evidence that has been unearthed regarding the inter-relationships between OSA and other comorbidities. Early and simple identification of risk markers can support the development of targeted therapies for management of OSA and its associated comorbidities [28].

Indriksone et al. conducted a systematic review on the existing literature in 2014 where they observed that insufficient evidence existed to conclude that the upper airway dimensions differed across various skeletal classes [29]. Again, this contrasts with the findings from this study in which the authors found that in individuals with skeletal Class II relations, the dimensions of the hypopharynx were found to be significantly smaller than in skeletal Class III patients. It was also observed that the dimensions of the nasopharynx were found to be smaller in skeletal Class III than skeletal Class II patients. This could be partly attributed to the fact that the present study utilised values obtained from CBCT records while most previous studies analysed cephalometric records which could report volumetric data precisely since these were 2-dimensional records. Zheng et al. conducted a three-dimensional evaluation of the upper airway in patients with different anterio-posterior skeletal patterns and found that the volume of the airway varied for different patterns [30]. They found that the nasopharyngeal airway was much larger in skeletal Class I and III patients compared to skeletal Class II individuals. This contrasts with the findings of the present study in which we observed that the nasopharyngeal airway was reduced in skeletal Class III when compared to Class I and II skeletal relations. In patients presenting with Class III malocclusions the mandibular overgrowth is frequently accompanied by maxillary hypoplasia [31]. Despite this finding there is very less literature on the relationship between skeletal Class III malocclusions and sleep apnea. Previous study has shown that the use of a combination of rapid maxillary expander and Delaire face mask can help increase the nasopharyngeal volume [32].

Orthodontic treatment relies upon the parameters studied in this manuscript, with Sella measurements and landmarks routinely used for diagnostic purposes and various classifications employ the use of this landmark to report the type of skeletal malocclusion present in particular cases. The identified skeletal malocclusions then directly influence orthodontic treatment planning, which in turn has been demonstrated to have an effect on the pharyngeal airway space. This is why these parameters were considered for this study so that some more light could be shed on their relationships and to offer a better understanding in terms of orthodontic diagnosis and treatment planning.

Our results must be interpreted with caution since the upper pharyngeal airway involves complex anatomical structures so there can be many variations present across different skeletal patterns, even though CBCTs are the most reliable option to conduct a three-dimensional analysis of the upper airway as has been demonstrated in previous studies [33, 34]. The major limitations of the present study are that it was a single centre study that utilised convenience sampling and only CBCTs recorded at the researchers’ university hospital were used. For future studies it is recommended to consider conducting a multi-centre study with sample groups further sub-divided based on their ethnic origin. Furthermore, multi-cultural studies could be conducted using the data from our study to better understand differences in the airway dimensions across skeletal Classes I, II, and III and skeletal relations from different regions.

## Conclusion

Consistent correlations between Sella turcica parameters and various facial skeletal profiles were revealed. Skeletal Class II individuals exhibited a distinct pattern and skeletal Class I and Class III demonstrated an average relationship. Airway volume discrepancies in nasopharynx and hypopharynx were observed in skeletal Class III and Class II individuals, respectively. There was no significant gender difference in all skeletal parameters. NCV demonstrated significant differences between skeletal Class I–Class III as well as between skeletal Class II–Class III. HCV also demonstrated significant differences between skeletal Class I–Class II and between skeletal Class II–Class III.

## Data Availability

Data are available from corresponding authors upon reasonable request.

## References

[1] King EW. A roentgenographic study of pharyngeal growth. Angle Orthod Angle Orthod. 1952;22(1):23–37.

[2] Tourne LP. Growth of the pharynx and its physiologic implications. Am J Orthod Dentofac Orthop. 1991;99(2):129–39. 10.1016/0889-5406(91)70115-D 1990822

[3] Khosla S, Caton N, Zhang TT, Davies-Husband CR. Parapharyngeal abscess secondary to lymphovenous malformation. J Laryngol Otol. 2019;133(3):256–9. 30821227 10.1017/S0022215119000318

[4] Forte AJ, Lu X, Hashim PW, Steinbacher DM, Alperovich M, Persing JA, Alonso N. Analysis of airway and midface in Crouzon syndromes. Ann Plast Surg. 2019;82(6):686–91. 30633021 10.1097/SAP.0000000000001740

[5] Momany SM, AlJamal G, Shugaa-Addin B, Khader YS. Cone beam computed tomography analysis of upper airway measurements in patients with obstructive sleep apnea. Am J Med Sci. 2016;352(4):376–84. 27776719 10.1016/j.amjms.2016.07.014

[6] Hirata RP, Aguiar IC, Nacif SR, Giannasi LC, Leitão Filho FS, Santos IR, Romano S, Faria NS, Nonaka PN, Sampaio LM, Oliveira CS. Observational study on efficacy of negative expiratory pressure test proposed as screening for obstructive sleep apnea syndrome among commercial interstate bus drivers-protocol study. BMC Pulm Med. 2011;7:11. 10.1186/1471-2466-11-57 PMC3280188 22151802

[7] Sériès F, Marc I. Nasal pressure recording in the diagnosis of sleep apnoea hypopnoea syndrome. Thorax. 1999;54(6):506–10. 10335004 10.1136/thx.54.6.506 PMC1745498

[8] Schwab RJ, Gupta KB, Gefter WB, Metzger LJ, Hoffman EA, Pack AI. Upper airway and soft tissue anatomy in normal subjects and patients with sleep-disordered breathing. Significance of the lateral pharyngeal walls. Am J Respir Crit Care Med. 1995;152(5 Pt 1):1673–89. 7582313 10.1164/ajrccm.152.5.7582313

[9] Stuck BA, Maurer JT. Airway evaluation in obstructive sleep apnea. Sleep Med Rev. 2008;12(6):411–36. 18054259 10.1016/j.smrv.2007.08.009

[10] Ghoneima A, Kula K. Accuracy and reliability of cone-beam computed tomography for airway volume analysis. Eur J Orthod. 2013;35(2):256–61. 21832270 10.1093/ejo/cjr099

[11] Scarfe WC, Farman AG, Sukovic P. Clinical applications of cone-beam computed tomography in dental practice. J Can Dent Assoc. 2006;72(1):75–80. 16480609

[12] Vos WD, De Backer J, Devolder A, Vanderveken O, Verhulst S, Salgado R, Germonpré P, Partoens B, Wuyts F, Parizel P, De Backer W. Correlation between severity of sleep apnea and upper airway morphology based on advanced anatomical and functional imaging. J Biomech. 2007;40(10):2207–13. 17178125 10.1016/j.jbiomech.2006.10.024

[13] Fang MR, Yan XZ, Ni JL, Gu YG, Meng L, Yuan LC, Cai HY, Wang LR, Qin JW, Cai Q, Zhang Y. Study of pharyngeal airway morphology with CBCT: benefits of four premolar extraction orthodontic treatments. Niger J Clin Pract. 2022;25(12):1955–62. 36537450 10.4103/njcp.njcp_1815_21

[14] Wang Q, Jia P, Anderson NK, Wang L, Lin J. Changes of pharyngeal airway size and hyoid bone position following orthodontic treatment of class I bimaxillary protrusion. Angle Orthod. 2012;82(1):115–21. 21793712 10.2319/011011-13.1 PMC8881045

[15] Schwab RJ, Goldberg AN. Upper airway assessment: radiographic and other imaging techniques. Otolaryngol Clin North Am. 1998;31(6):931–68. 9838010 10.1016/s0030-6665(05)70100-6

[16] Metes A, Hoffstein V, Direnfeld V, Chapnik JS, Zamel N. Three-dimensional CT reconstruction and volume measurements of the pharyngeal airway before and after maxillofacial surgery in obstructive sleep apnea. J Otolaryngol. 1993;22(4):261–4. 8230377

[17] Abdulhameed SA, Riyaz Ss MA, Almutairy M, Khan NS, Jayakumar S, Gaonkar P. Assessing the accuracy of lateral cephalogram in quantifying three-dimensional pharyngeal airway morphology compared to Cone-Beam Computed Tomography. Cureus. 2024;16(3):e57301. 38690459 10.7759/cureus.57301 PMC11059114

[18] Bronoosh P, Khojastepour L. Analysis of pharyngeal Airway using lateral cephalogram vs CBCT images: a cross-sectional retrospective study. Open Dent J. 2015;31:9:263–6. 10.2174/1874210601509010263 PMC4598371 26464593

[19] Abé-Nickler MD, Pörtner S, Sieg P, Hakim SG. No correlation between two-dimensional measurements and three-dimensional configuration of the pharyngeal upper airway space in cone-beam computed tomography. J Craniomaxillofac Surg. 2017;45(3):371–6. 28187974 10.1016/j.jcms.2017.01.004

[20] Muto T, Yamazaki A, Takeda S. A cephalometric evaluation of the pharyngeal airway space in patients with mandibular retrognathia and prognathia, and normal subjects. Int J Oral Maxillofac Surg. 2008;37(3):228–31. 18296029 10.1016/j.ijom.2007.06.020

[21] Fedorov A, Beichel R, Kalpathy-Cramer J, Finet J, Fillion-Robin J-C, Pujol S, Bauer C, Jennings D, Fennessy FM, Sonka M, Buatti J, Aylward SR, Miller JV, Pieper S, Kikinis R. 3D slicer as an image Computing platform for the Quantitative Imaging Network. Magn Reson Imaging. 2012;30(9):1323–41. 22770690 10.1016/j.mri.2012.05.001 PMC3466397

[22] El H, Palomo JM. An airway study of different maxillary and mandibular sagittal positions. Eur J Orthod. 2011;35(2):262–70. 22045695 10.1093/ejo/cjr114

[23] Soni J, Shyagali TR, Bhayya DP, Shah R. Evaluation of pharyngeal space in different combinations of class II skeletal malocclusion. Acta Inf Med. 2015;23(5):285–9. 10.5455/aim.2015.23.285-289 PMC4639349 26635436

[24] Oz U, Orhan K, Rubenduz M. 2D lateral cephalometric evaluation of varying types of class II subgroups on posterior airway space in postadolescent girls: a pilot study. J Orofac Orthop. 2013;74(1):18–27. 23307178 10.1007/s00056-012-0121-0

[25] Ceylan I, Oktay H. A study on the pharyngeal size in different skeletal patterns. Am J Orthod Dentofac Orthop. 1995;108(1):69–75. 10.1016/s0889-5406(95)70068-4 7598107

[26] Chokotiya H, Banthia A, Srinivasa RK, Choudhary K, Sharma P, Awasthi N. A study on the evaluation of pharyngeal size in different skeletal patterns: a Radiographic Study. J Contemp Dent Pract. 2018;19(10):1278–83. 30498186

[27] Kezirian EJ, Goldberg AN. Hypopharyngeal surgery in obstructive sleep apnea: an evidence-based Medicine Review. Arch Otolaryngol Head Neck Surg. 2006;132(2):206–13. 16490881 10.1001/archotol.132.2.206

[28] McNicholas WT, Pevernagie D. Obstructive sleep apnea: transition from pathophysiology to an integrative disease model. J Sleep Res. 2022;31(4):e13616. 35609941 10.1111/jsr.13616 PMC9539471

[29] Indriksone I, Jakobsone G. The upper airway dimensions in different sagittal craniofacial patterns: a systematic review. Stomatologija. 2014;16(3):109–17. 25471995

[30] Zheng ZH, Yamaguchi T, Kurihara A, Li HF, Maki K. Three-dimensional evaluation of upper airway in patients with different anteroposterior skeletal patterns. Orthod Craniofac Res. 2014;17(1):38–48. 24033888 10.1111/ocr.12029

[31] Pavoni C, Cretella Lombardo E, Lione R, Bollero P, Ottaviani F, Cozza P. Orthopaedic treatment effects of functional therapy on the sagittal pharyngeal dimensions in subjects with sleep-disordered breathing and class II malocclusion. Acta Otorhinolaryngol Ital. 2017;37:479–85. 10.14639/0392-100X-1420. 29327733 10.14639/0392-100X-1420 PMC5782425

[32] Caruso S, Lisciotto E, Caruso S, Marino A, Fiasca F, Buttarazzi M, Sarzi Amadè D, Evangelisti M, Mattei A, Gatto R. Effects of Rapid Maxillary Expander and Delaire Mask Treatment on Airway Sagittal Dimensions in Pediatric patients affected by Class III Malocclusion and Obstructive Sleep Apnea Syndrome. Life (Basel). 2023;13(3):673. 36983829 10.3390/life13030673 PMC10056418

[33] Edwards R, Alsufyani N, Heo G, Flores-Mir C. The frequency and nature of incidental findings in large-field cone beam computed tomography scans of an orthodontic sample. Prog Orthod. 2014;15(1):37. 25033888 10.1186/s40510-014-0037-x PMC4884029

[34] Horner K, Islam M, Flygare L, Tsiklakis K, Whaites E. Basic principles for use of dental cone beam computed tomography: Consensus guidelines of the European Academy of Dental and Maxillofacial Radiology. Dentomaxillofac Radiol. 2009;38(4):187–95. 19372107 10.1259/dmfr/74941012

